# Temperature Dependence of the Vibrational Wave-Packet Dynamics of Cu_5_

**DOI:** 10.1021/acs.jpca.5c06058

**Published:** 2025-11-18

**Authors:** Jia Han, Björn Bastian, Marcel Jorewitz, Knut R. Asmis, Jiaye Jin

**Affiliations:** Wilhelm-Ostwald-Institut für Physikalische und Theoretische Chemie, 9180 Universität Leipzig, Linnéstr. 2, 04103 Leipzig, Germany

## Abstract

Vibrational wave-packet dynamics on the electronic ground state of the neutral copper pentamer (Cu_5_) are studied by femtosecond (fs) pump–probe spectroscopy using the ‘negative ion to neutral to positive ion’ excitation scheme (NeNePo). A vibrational wave packet is prepared on the electronic ground state (^2^A_1_) of Cu_5_ via photodetachment of a mass-selected, cryogenically cooled Cu_5_^–^ anion using the first fs pump pulse. The temporal evolution of the vibrational wave packet is then probed by a second ultrafast probe pulse via resonant multiphoton ionization to Cu_5_^+^. A frequency analysis of the femtosecond NeNePo transients for pump–probe delay times from 0.2 to 20.0 ps reveals two primary beating frequencies at 148 and 108 cm^–1^ as well as weak and transient frequency features at 222, 216, 76, and 40 cm^–1^. A comparison of experimentally obtained beating frequencies to the harmonic frequencies of normal modes obtained from quantum chemistry calculations confirms that Cu_5_ in the gas phase adopts a planar trapezoidal geometry. NeNePo transients measured at ion-trap temperatures from 20 to 270 K probe the influence of the ion temperature on the wave-packet dynamics obtained. The inverse correlation between the oscillation lifetime τ_1/2_ and the square root of the temperature indicates a vibrational decoherence channel originating from the anharmonicity of high-energy vibrational levels.

## Graphical abstract

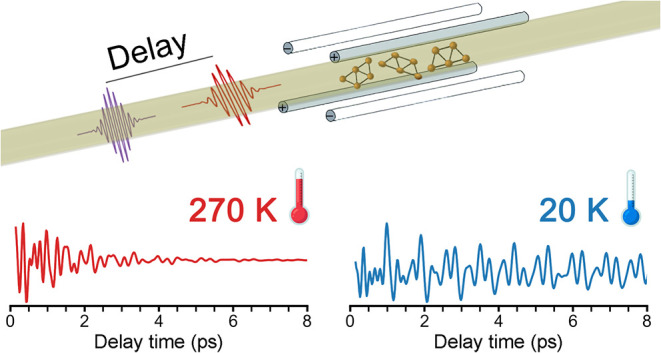

## 1 Introduction

Copper clusters have received considerable attention due to their remarkable structural and electronic properties,^1–4^ which are highly sensitive to environmental perturbations and temperature fluctuations. Understanding their structural fluxionality and relaxation dynamics among geometric isomers is essential for elucidating the reaction mechanisms relevant in heterogeneous catalysis, guiding the rational design of nanomaterials, and uncovering the fundamental nature of bonding in small aggregates.^4–8^

The structural fluxionality of small copper clusters has been widely investigated, particularly for supported clusters and those at high temperatures.^9–15^ One key feature is the interconversion between different isomers, such as planar and three-dimensional (3D) structures, which strongly influences the preferred adsorption sites and the reactivity toward small molecules. The copper pentamer, Cu_5_, exhibits two distinct isomers, which are shown in Scheme 1. Planar structure **1** is calculated to be ≈19 kJ/mol more stable than the bipyramidal structure **2** using coupled-cluster theory,^15^ considerably lower than that for Ag_5_ (≈41 kJ/mol).^16^ Cu_5_ **1** has been synthesized in solution and exhibits high stability.^17^ However, the reactivity of **1** is much lower than that of **2**, which has been found on the surfaces and predicted to be more fluxional and more reactive toward molecular oxygen.^9–13,18^ Cu_5_ therefore serves as a model system to investigate the interconversion and fluxional properties of small metal clusters to obtain a better understanding of the intrinsic connection between reactivity and structural fluxionality.

**Scheme 1:**
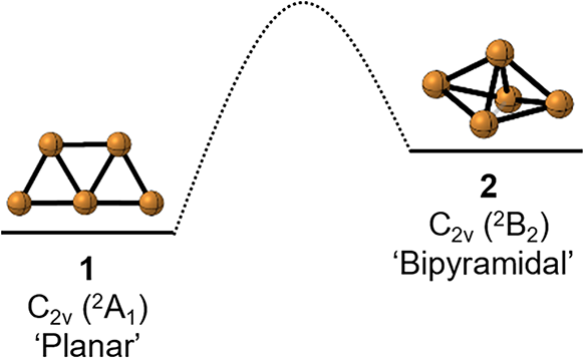
Structures of Cu_5_: Planar **1** and Bipyramidal **2**

Gas-phase spectroscopy of mass-selected clusters avoids environmental perturbations, which has proven very useful for understanding these dynamic properties.^19–25^ A different energetic ordering of Cu_5_ isomers has also been found in the gas phase and in cryogenic matrices. Bipyramidal Cu_5_ **2** was first identified in a cyclohexane matrix at 77 K by using electron spin resonance spectroscopy and theoretical calculations.^26^ Infrared photodissociation spectroscopy of Cu_5_^+^(Ar)_3_ provided evidence for a bipyramidal geometry of Cu_5_^+^.^27^ However, a further study of Cu_5_^+^(He)_4_ predicted a twisted X-structure based on density functional theory (DFT) calculations.^28^ Moreover, the infrared multiple photon dissociation spectra of [Cu_5_,2H/2D]^+^ agreed well with the predicted spectrum of yet another 3D structure containing a tetrahedron Cu_4_ motif capped by a bridging Cu.^29^ Several groups have reported on the anion photoelectron spectroscopy (PES) of Cu_5_^–^, but without achieving vibrational resolution.^30–34^ The obtained vertical detachment energy (VDE) of ≈1.94 eV was assigned to the transition from the anionic electronic ground state (^1^A_1_) to the neutral electronic ground state (^2^A_1_) of the planar trapezoidal structure **1** based on quantum chemistry calculations. Optical absorption spectra of the neutral copper pentamer were studied in different solid matrices,^35–37^ confirming the assignment of the planar structure **1** to the neutral electronic ground state of Cu_5_.

To address the fluxionality of Cu_5_ in a specific charge state and under well-controlled conditions, it proves helpful to combine mass-selective methods with temperature control and temporal resolution to capture nuclear motions. This can be achieved by using a temperature-controlled cryogenic ion trap, which stores ions long enough for them to thermalize close to the ambient temperature and allows subsequent modulation using laser excitation. Cryo-SEVI is a comprehensive method for obtaining vibrational information of neutral metal clusters by measuring the kinetic energies of slow photoelectrons detached from corresponding, vibrationally cold anions, which were initially stored in a cryogenic ion trap.^23^ Femtosecond (fs) negative ion to neutral to positive ion (NeNePo) spectroscopy represents an alternative method for studying the vibrational wave-packet dynamics of neutral metal clusters, prepared by photodetachment of the corresponding anions in a cryogenically cooled ion trap.^38–45^ The observed oscillatory signals originate from the quantum beating of vibrational wave packets, reflecting the vibrational frequencies of the neutral clusters. Using this method, we have obtained vibrational constants of the ground electronic state (X^1^A_2_) of the silver dimer^43^ and excited electronic states of the cerium dimer.^45^ Additionally, vibrational wave-packet dynamics obtained using fs NeNePo spectroscopy have confirmed a planar trapezoidal structure of Ag_5_.^44^

In this work, we employ temperature-dependent fs NeNePo spectroscopy to investigate the vibrational wave-packet dynamics of Cu_5_ prepared by photodetachment from mass-selected Cu_5_^–^ at different ion-trap temperatures ranging from 20 to 270 K. At the lowest temperature, oscillations in cation yield are observed over several picoseconds. These are attributed to the vibrational wave-packet dynamics on the neutral electronic ground state involving the excitation of four totally symmetric vibrational modes, consistent with planar structure **1**. No evidence of the bipyramidal structure **2** is observed in fs NeNePo transients, also not at higher ion-trap temperatures. We examine the influence of the ion temperature on the vibrational wave-packet dynamics, demonstrating the applicability of fs NeNePo spectroscopy for investigating the structural dynamics in neutral metal clusters over a broad temperature range.

## 2 Methods

### 2.1 Experimental Methods

The fs NeNePo transients were measured using a tandem mass spectrometer with an integrated, cryogenically cooled linear quadrupole ion trap and a pulsed Ti:Sapphire femtosecond laser system, as described previously.^43,44^ Briefly, Cu_5_^–^ anions are produced by aggregation using a copper target (99.999%, Kurt J. Lesker) as the anode of a direct current (DC) magnetron sputtering source (TORUS, Kurt J. Lesker) at liquid nitrogen temperature. The most abundant natural isotopologue of Cu_5_^+^, ^63^Cu_4_^65^Cu (*m*/*z* = 317), is mass-selected and continuously accumulated in a cryogenic ion trap filled with 0.2–0.3 mbar helium buffer gas. The ion trap is directly attached to the second stage of a closed-cycle helium cryostat (Sumitomo SRDK-408E2, F50H compressor). The trap temperature is controlled using a Cernox sensor and heating cartridge connected to a Lake Shore Model 335 temperature controller. Collisions with the helium buffer gas thermalize the trapped anions close to the trap temperature, which can be held at temperatures ranging from 15 to 300 K.

Two fs laser pulses, referred to as the pump pulse and probe pulse, are collinearly combined, propagate along the trap axis, and are focused near the center of the ion trap through a 1.5 mm thick CaF_2_ lens (*f* = +1000 mm). The excitation scheme is given in Figure 1. A vibrational wave packet in Cu_5_ is launched with the first vertically polarized fs laser pulse (autocorrelation duration of 65 fs, ≈4 μJ/pulse) by electron photodetachment of cold Cu_5_^–^. The polarization of the probe pulse is adjusted between parallel (θ = 0°) and perpendicular (θ = 90°) with respect to that of the pump pulse by a MgF_2_ λ/2 waveplate (EKSMA). Two different pump wavelengths, one centered at 585 nm (2.12 eV) and the other at 602 nm (2.06 eV), are applied in the present experiments. Considering the vertical detachment energy (1.94 eV) of Cu_5_^–^,^30–34^ single-photon detachment is possible at both wavelengths, but both remain far below the excitation energy to the first accessible excited electronic state of Cu_5_ located at 3.14 eV above the anion ground state.^31^ The temporal evolution of the vibrational wave-packet dynamics of Cu_5_ is then probed using a second ultrafast laser pulse (centered at 406 nm, autocorrelation duration of 50 fs, 1.5 μJ/pulse), at the magic angle (θ = 55°) to avoid rotational dynamics, ionizing Cu_5_ by resonant ionization (ionization potential ≈6.3 eV).^37,46^ Cu_5_^+^ exhibits broad photodissociation absorption bands from 500 to 370 nm.^47^ Fragments of Cu_5_^+^ are produced under irradiation of the probe pulse. The resulting cations, including cationic fragments, are no longer confined along the trap axis, expelled from the ion trap in the axial direction, and subsequently analyzed by a second quadrupole mass filter. Fs NeNePo transients are obtained by monitoring the mass-selected cation yield for 2000 ms accumulation time as a function of the delay time with a step of 20 fs.

**Figure 1:**
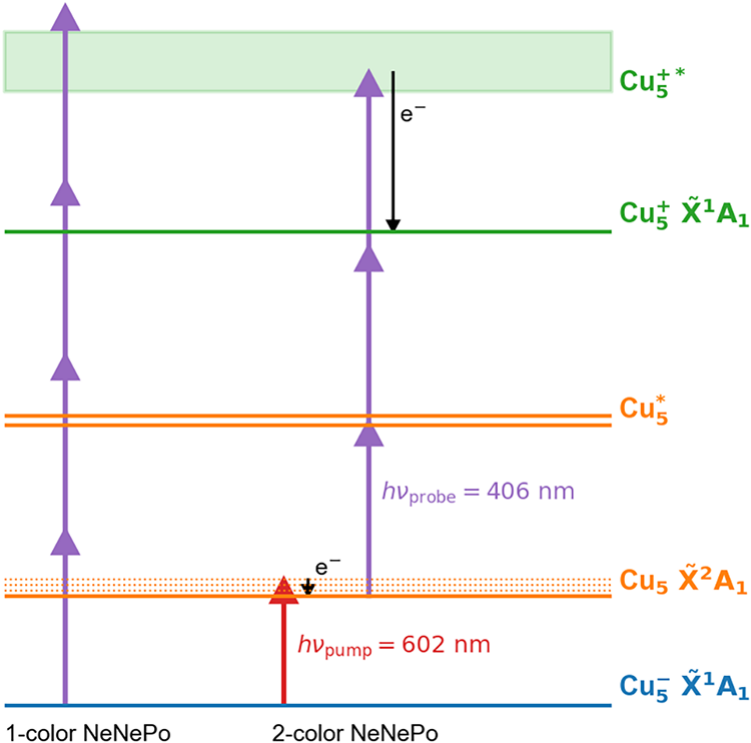
Fs NeNePo pump–probe excitation scheme for Cu_5_ over three charge states (blue: anion; orange: neutral; green: cation).

The frequency content of the oscillatory component of the fs NeNePo transient is obtained by computing its Fourier transform (FT). The high-frequency oscillatory signal is extracted by removing the low-frequency contributions using the symmetric least-squares method.^48,49^ Then, a Kaiser window function (α = 4.5) is used to balance the main-lobe width and side-lobe level. Zero values are padded to the oscillation parts up to 2 times the data length, in order to minimize the influence of a nonintegral cut of the periods. Additionally, short-time window Fourier transform (STFT) spectrograms are computed to analyze frequency components using a sliding window time of 5.5 ps, providing a frequency resolution of 6 cm^–1^.

The oscillatory signal is also fitted to a sum of cosine functions containing all significant frequency contributions, as $f\left(t\right)=\underset{i}{\sum }A_{i}\;\cos\left(\frac{2\pi t}{T_{i}}+\phi_{i}\right)e^{-t/\tau_{i}}$ , where *A*_*i*_ is the oscillation amplitude, τ*_i_* is the dephasing lifetime, *T_i_* is the period of the oscillation, and ϕ_*i*_ is the initial phase of the oscillation, in order to study the initial phases and the coherence time of each frequency component. Similar to our previous work on Ag_5_,^44^ the initial amplitudes *A*_*i*_, oscillation periods *T_i_* (or frequencies, 1/(ω_e_ *c*)), and lifetimes τ*_i_* are taken from the STFT spectrogram, and the initial phases ϕ_*i*_ are set to 0.

### 2.2 Computational Methods

Geometry optimizations and frequency calculations were performed using the GAUSSIAN16 C.01 program.^50^ Geometries were optimized using DFT employing with the PBE0 functional^51,52^ with a triple-ζ basis set (def2-TZVPP)^53,54^ and a superfine grid (pruned 250,974 for Cu atom). The harmonic vibrational frequencies were obtained from analytical second derivatives using the same method. The PBE0 functional has been shown to reliably reproduce basic properties of small metal clusters.^55^ The vibrational-level population is calculated from Franck–Condon factors within the parallel approximation using the ezFCF software package.^56^

## 3 Results and Discussion

### 3.1 Fragmentation and fs NeNePo Transients

Quadrupole mass spectra of Cu_*n*_^+^ (*n* = 1–5) were measured by irradiating mass-selected Cu_5_^–^ anions, trapped in the ion trap held at 20 K, with two fs pulses (λ_pump_ = 585 nm and λ_probe_ = 406 nm). Figure S1 shows integrated cation counts for a delay time at −200 fs (Figure S1A) and at +200 fs (Figure S1B). Under these conditions, the ion yield of Cu_5_^+^ is lower than that of the fragment, Cu_3_^+^, while other fragment ions, such as Cu_2_^+^ and Cu_4_^+^, are only observed as minor contributions. This is different from our previous results on Ag_5_^–^, where Ag_5_^+^ is observed as the most abundant cation.^44^ Laser power dependence measurements of the cation yields obtained using only the probe pulse (Figures S2A and S3) reveal that three photons are required to ionize Cu_5_^–^ to Cu_5_^+^, whereas an additional photon is needed to form Cu_3_^+^. The same trend is evident in the laser power dependence performed at slightly longer pump wavelengths (λ_pump_ = 602 nm and λ_probe_ = 406 nm) at +960 fs delay time (Figures S2B and S4). These findings agree with the proposed excitation scheme (Scheme 1) and confirm that Cu_3_^+^ is produced from the photodissociation of Cu_5_^+^ by the probe pulse.^47^

The fs NeNePo transients of Cu_5_ obtained for λ_pump_ = 602 nm (5 μJ/pulse) and λ_probe_ = 406 nm (2 μJ/pulse) by monitoring Cu_5_^+^ and Cu_3_^+^ in a delay time range from −1 to +4 ps, are shown in Figure 2. At this laser power, both transients show low ion counts without clear oscillations at negative delay times. A gradual rise of the cation yield in both cation channels is observed as the delay time increases from a negative delay time. The Cu_5_^+^ signal shows a maximum at zero delay time followed by a rapid decay within ≈1 ps and negligible oscillations, while a clear oscillatory signal is observed in the Cu_3_^+^ channel. Excited electronic states of Cu_5_ are likely populated by the high laser power applied in the present study. These states then undergo a rapid relaxation that extends beyond the cross-correlation duration of the laser pulses. This is consistent with recently reported ultrafast dynamics of neutral metal clusters, which show comparable behaviors.^57–59^ We note that the transient in Cu_5_^+^ also shows decay within ≈1 ps in the negative delay time region, which may result from the dynamics induced by the 406 nm pulse and then probed by the 602 nm pulse. This is notably different from those reported in our recent study of the vibrational wave-packet dynamics of Ag_5_. This may arise from higher laser power applied in the current study as well as slightly different Franck–Condon factors. The transient observed in the Cu_5_^+^ channel is similar to that observed in previous fs NeNePo study for Ag_5_, where more than 30 μJ/pulse was used.^60^ In contrast, our recent work reported clear oscillations in the Ag_5_^+^ channel using much lower pulses power of ≈0.5 μJ/pulse.^44^ Under such conditions, the total Cu_*n*_^+^ counts are significantly reduced (see Figures S2–S4), likely due to the lower overall photoionization cross sections of copper clusters compared to those of silver clusters.^61^ The maximum cation yield of Cu_3_^+^ is delayed by 80 fs relative to that of Cu_5_^+^, within the temporal width of the cross-correlation of two laser pulses. Therefore, Cu_3_^+^ and Cu_5_^+^ are generated nearly simultaneously. The formation of Cu_3_^+^ likely arises from photodissociation of electronically excited states of Cu_5_^+^, rather than from a relaxed excited state of Cu_5_ or Cu_5_^–^, which typically have a longer time delay due to geometric relaxation.

**Figure 2:**
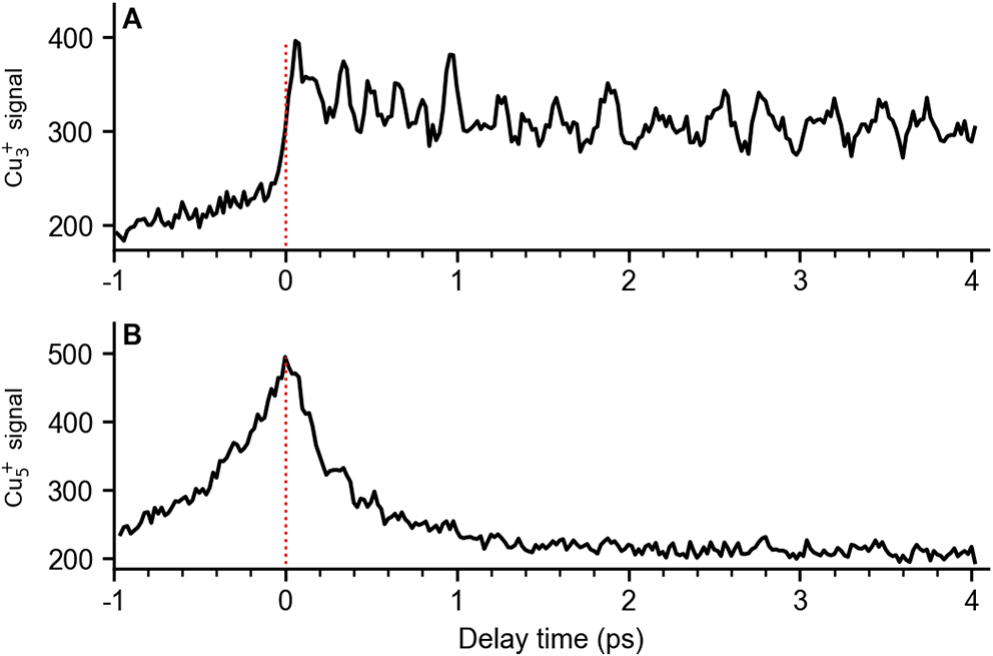
Fs NeNePo transients for Cu_5_ recorded at λ_pump_ = 602 nm (5 μJ/pulse) and λ_probe_ = 406 nm (2 μJ/pulse), monitoring Cu_3_^+^ (A) and Cu_5_^+^ (B) over delay times from −1 to +4 ps with a step of 20 fs. The ion trap is filled with around 0.3 mbar of helium and held at 20 K. The polarization of the probe pulse is set to 55°, with respect to the polarization of the pump pulse. The zero delay time is indicated by a red dotted line.

### 3.2 Frequency Analysis and Structure Determination

In the following, we focus on the oscillatory signal observed in the Cu_3_^+^ channel for λ_probe_ = 406 nm. The fs NeNePo transients were recorded for two different pump pulse wavelengths centered at 585 and 602 nm, shown in Figures 3 and S5, respectively. The fs NeNePo transient obtained using λ_pump_ = 585 nm (Figure 3A) shows a clearer oscillatory Cu_3_^+^ yield at positive delay time up until 20 ps, compared to the transient obtained using λ_pump_ = 602 nm (Figure S5A), likely due to more efficient photodetachment using higher photon energy. It should be noted that the photon energy of the 602 nm pulse (2.06 eV) is close to the VDE of Cu_5_^–^ (1.94 eV). Hence, such a small energy shift to 585 nm (2.12 eV) may substantially increase the photodetachment probability.^62,63^ The observed oscillations do not reflect only a single frequency, suggesting that multiple frequency components correspond to the excitation of several vibrational modes and contribute to the wave-packet dynamics. The oscillations continue up to ≈15 ps and eventually decay to a constant (nonoscillatory) cation yield level substantially higher than that observed at negative delay time. Recurrence of the oscillations is not found up to 40 ps. This may result from vibrational anharmonicities, leading to a fast dephasing and a longer time required for the revival of the wave packet. Alternatively, it may also arise from efficient coupling to the intramolecular “bath” states leading to intramolecular vibrational energy redistribution (IVR) and consequently vibrational decoherence.

**Figure 3:**
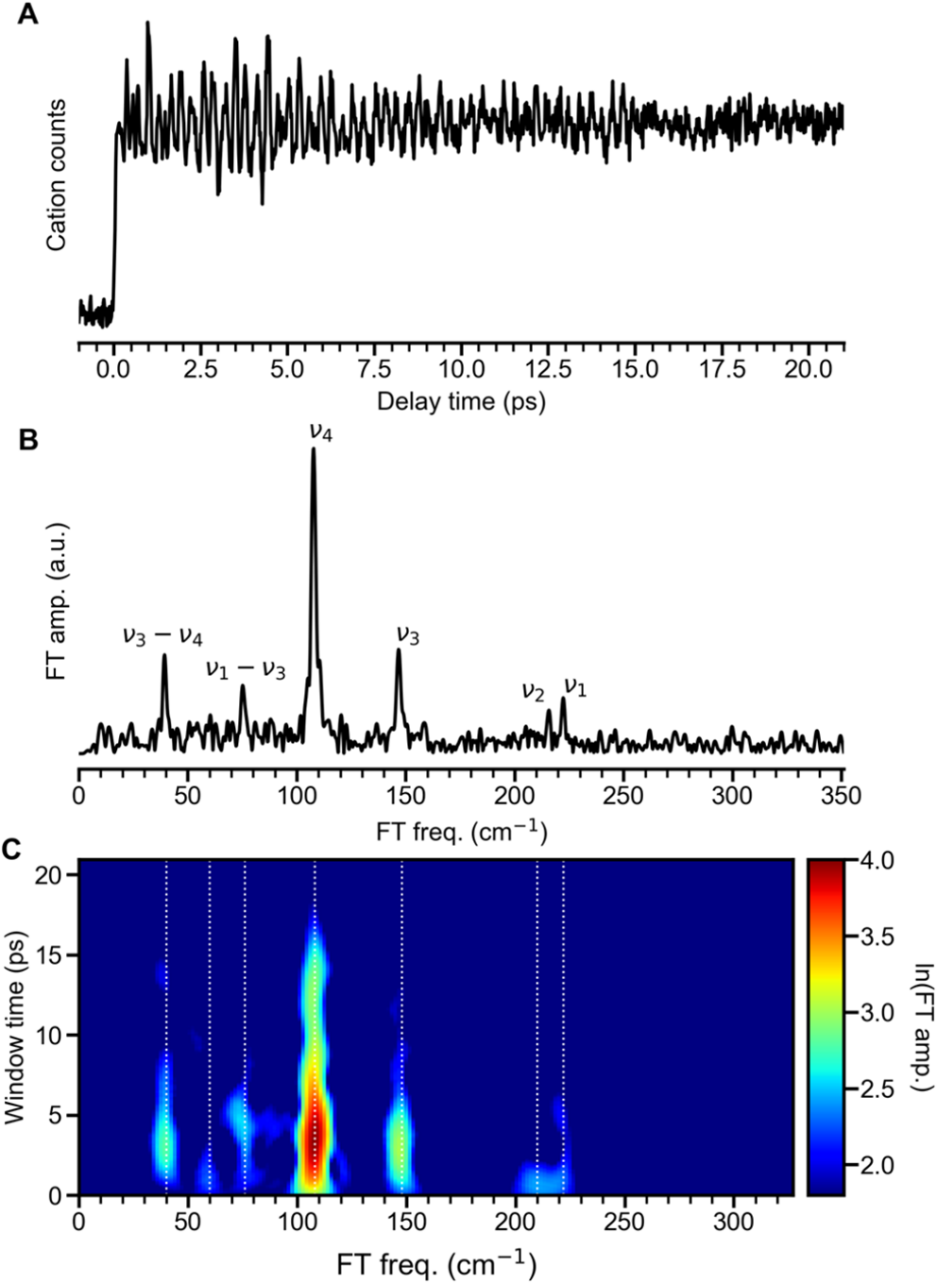
(A) fs NeNePo transients over a delay time range from −1 to +21 ps (λ_pump_ = 585 nm, λ_probe_ = 406 nm, and θ = 55°). (B) FT amplitude spectrum for extracted oscillatory signals and (C) STFT spectrogram obtained by applying a 5.5 ps window time providing a frequency resolution of 6 cm^–1^. The observed frequencies (white dotted lines) and the displacements of involved vibrational modes are given in Table 1 and Figure 4, respectively.

The oscillatory part of the transients is extracted from 0.1 to 20 ps for FT analysis. The resulting FT amplitude spectra are given in Figures 3B and S5B, respectively, showing a frequency resolution of 1.7 cm^–1^. The FT spectrum with λ_pump_ = 585 nm (Figure 3B) exhibits a dominant band centered at 108 cm^–1^. Additional weaker features below 300 cm^–1^ centered at 222, 216, 148, 76 and 40 cm^–1^ are also observed. By contrast, only two bands centered at 148 and 108 cm^–1^ are clearly resolved in the spectrum with λ_pump_ = 602 nm (Figure S5B), likely reflecting the better signal-to-noise ratio of the oscillatory cation yield using λ_pump_ = 585 nm. The common occurrence of the frequency bands at 148 and 108 cm^–1^ suggests vibrational wave-packet dynamics on the same electronic state, which we assign to the electronic ground state of Cu_5_, previously characterized by vibrationally unresolved anion photoelectron spectra. Additionally, we determined STFT spectrograms using a sliding window time of 5.5 ps to reveal weak and transient features. This provides a frequency resolution of 6 cm^–1^. The most prominent oscillation features in the STFT spectrogram (Figure 3C) correspond to the frequency band at 108 cm^–1^, which persists for ≈16 ps, while features at different frequencies are shorter-lived and decay within 5 ps. Surprisingly, a weaker feature at 60 cm^–1^ is even shorter-lived (<2 ps) and evident in the STFT spectrogram. The temporal profiles of these modes extracted from the STFT spectrogram are shown in Figure S6.

The frequencies observed in the FT spectra are summarized in Table 1 along with the harmonic frequencies of two Cu_5_ isomers, **1** and **2** (see Scheme 1), calculated using DFT calculations (PBE0/def2-TZVPP). As we noted previously, only totally symmetric vibrational modes are typically excited upon photodetachment, and therefore contribute to the vibrational wave-packet dynamics probed by fs NeNePo spectroscopy.^43,44^ The frequencies of four totally symmetric modes of **1** are predicted to be at 213, 201, 139, and 100 cm^–1^ (Table 1), which agree well with FT frequencies at 222, 216, 148, and 108 cm^–1^, respectively. It is surprising that all four totally symmetric modes are detected. The dominant totally symmetric mode involved in vibrational wave-packet dynamics is the in-plane bending modes (ν_4_) of the Cu–Cu–Cu moiety (see Figure 4). The other weaker features are the 5-membered ring breathing mode (ν_1_), the stretching mode (ν_2_) of the Cu–Cu moiety, and the symmetric stretching (ν_3_) of the Cu–Cu–Cu moiety. Furthermore, the FT bands at 76, 60, and 40 cm^–1^ arise from the difference frequency of ν_1_–ν_3_, ν_2_–ν_3_, and ν_3_–ν_4_, respectively. These observed difference frequency bands indicate the presence of coherent coupling between vibrational modes, yielding a relatively strong vibrational coherence from the superpositions of these vibrational modes, as was reported previously for Ag_5_ and other trapped ions.^44,64–68^ The calculated frequencies of the totally symmetric vibrational modes for planar Cu_5_^–^ (Table S1) show less agreement with the observed frequencies. Therefore, the observation of dynamics originating from the anion can be excluded.

**Table 1:** Experimental FT Frequencies and PBE0/def2-TZVPP Harmonic Frequencies (in cm^–1^) of Totally Symmetric Vibrational Modes of Two Cu_5_ Isomers Shown in Scheme 1 ^a^

	**1**		**2**	
Exp.	Harm.	Ass.	Harm.	Ass.
			256	ν_1_(a_1_)
222	213	ν_1_(a_1_)		
216	201	ν_2_(a_1_)/2ν_4_		
148	139	ν_3_(a_1_)	158	ν_2_(a_1_)
			130	ν_3_(a_1_)
108	100	ν_4_(a_1_)		
76		ν_1_–ν_3_	87	ν_4_(a_1_)
60^b^		ν_2_–ν_3_		
40		ν_3_–ν_4_		

a The normal modes are shown in Figures 4, S7, and S8.

b Observed in the STFT spectrogram in the window time range from 0.1 to 3 ps, shown in Figure 3.

**Figure 4:**
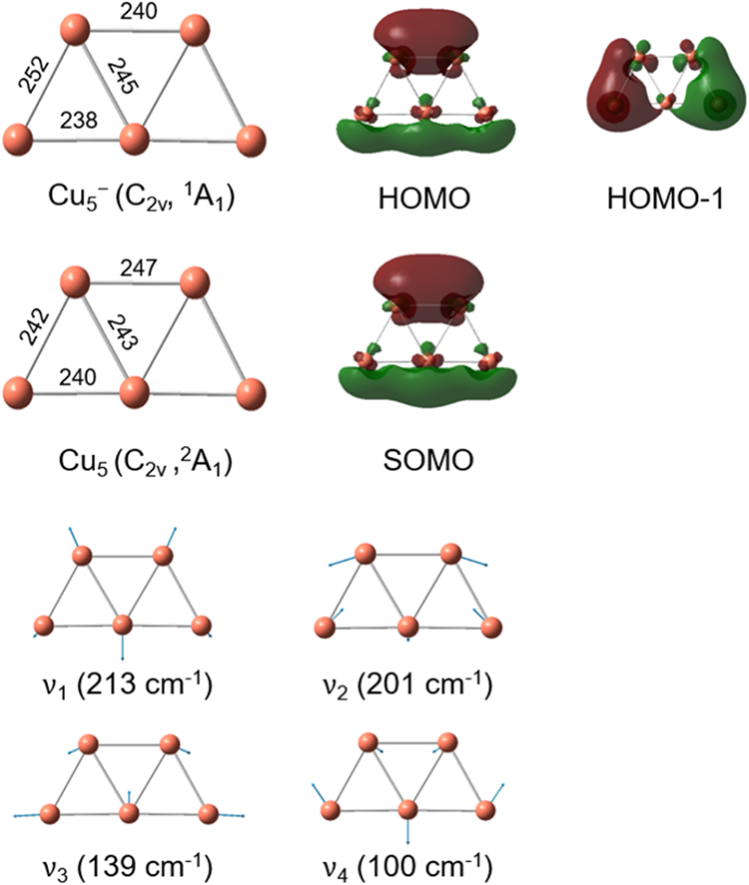
PBE0/def2-TZVPP minimum-energy geometries of Cu_5_ and Cu_5_^–^, singly occupied molecular orbital (SOMO) of Cu_5_, highest occupied molecular orbital (HOMO) of Cu_5_^–^, and involved harmonic vibrational mode displacements (see Figure S7 for the displacements of all modes and Table 1 for the harmonic frequencies). The Cu–Cu bond lengths are given in pm for Cu_5_ and Cu_5_^–^.

To identify which vibrational modes can be excited upon photodetachment, we computed the populations of vibrational levels determined from Franck–Condon factors at 20 K for the photodetachment transition, Cu_5_ (^2^A_1_,C_2v_) ← Cu_5_^–^ (^1^A_1_,C_2v_), as given in Figure S9B. Among these, the ν_2_ mode is predicted to be the most populated vibrationally excited state. Other totally symmetric modes, ν_1_, ν_3_, and ν_4_, are also excited, along with their high-quanta states, overtones and combinations. ν_5_ and ν_6_ are also excited at 20 K. Therefore, we cannot rule out that the 40 cm^–1^ FT band includes a contribution from nontotally symmetric modes resulting from vibrationally hot anions. These results are in good agreement with our previous analysis of beating frequencies, although the relative intensities notably differ from those in the FT spectrum, indicating that the excitation induced by the probe pulse may play an important role in modulating the observed beating frequencies.

The bipyramidal Cu_5_^–^ is calculated to be 19 kJ/mol higher than the planar anion using PBE0/def2-TZVPP, in agreement with previous theory results.^15,18,69,70^ In contrast to the calculated frequencies of **1**, the vibrational frequencies for the totally symmetric modes of **2** are centered at 256, 158, 130, and 87 cm^–1^ (see Table 1 and Figure S8), showing poorer agreement with the FT spectrum. Similarly, the predicted population for the vibrational modes of **2** at 20 K are less consistent with the experiments (see Figure S9). Therefore, the present results confirm the assignment of structure **1** in its electronic ground state.

The singly occupied molecular orbital (SOMO) of planar structure **1** and the highest occupied molecular orbital (HOMO) of Cu_5_^–^ shown in Figure 4 exhibit a nodal plane, which separates the upper Cu–Cu and lower linear Cu–Cu–Cu moieties. The geometric changes of Cu_5_ induced by the detachment of the photoelectron from the HOMO of Cu_5_^–^ mainly arise from the changes of the delocalization of the Cu_5_ SOMO compared to that of the Cu_5_^–^ HOMO (see Figure 4). The increases in bond lengths within the Cu–Cu and the Cu–Cu–Cu moieties, as well as the decrease between the two moieties, result in the excitation of the ring breathing mode ν_1_ and the symmetric stretching mode ν_3_. It should be noted that the photoionization and photofragmentation can provide additional selections on the observed wave-packet dynamics. The contributions from the Cu_3_^+^ channel may also lead to discrepancies between experimentally observed vibrational modes and the modes governed by a single Franck–Condon of photodetachment process.

### 3.3 Temperature Dependency of Vibrational Wave-Packet Dynamics

In order to investigate the influence of ion internal temperature on the vibrational wave-packet dynamics, we additionally measured the fs NeNePo transients at higher ion-trap temperatures between 20 and 270 K (see Figure S10). Collisions with the helium buffer gas in the ion trap thermalize the anions near the trap temperature, enabling us to study the ultrafast dynamics of isolated clusters at well-controlled temperatures. The overall transients measured at higher temperatures show more obvious decay from excited electronic neutral states within 1 ps, compared to that obtained at 20 K. The oscillatory ion yields extracted from 0.2 to 10 ps and the corresponding FT spectra are given in Figure 5. The transient features measured at higher temperatures are similar to those at 20 K. The ion yield remains constantly low at negative delay times, while a rise is observed at time zero, followed by oscillatory features. However, the oscillations decay faster with increasing temperature (see the left panel in Figure 5).

**Figure 5:**
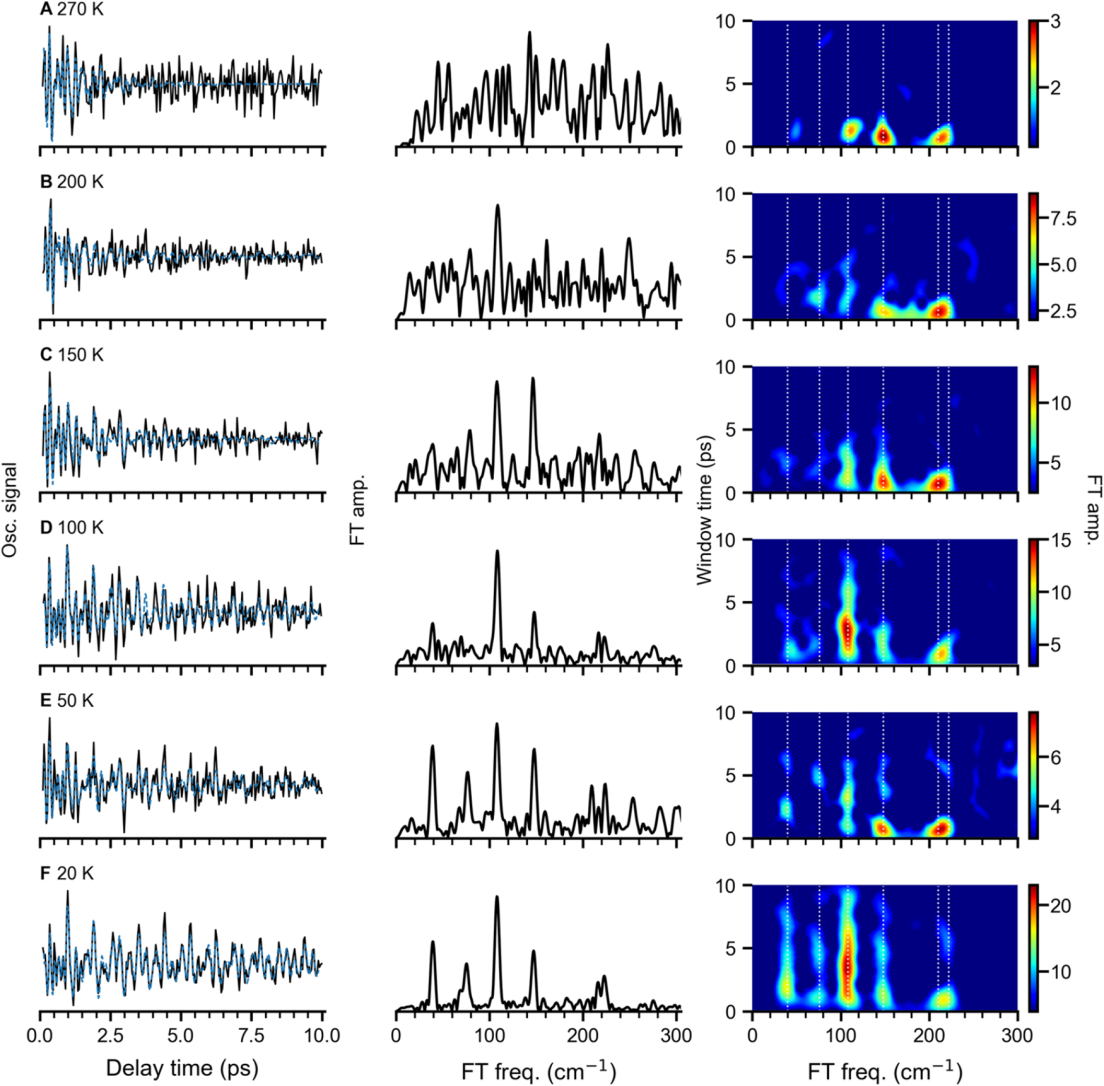
Oscillatory cation yields (left) over a delay time range from 0.2 to 10 ps (λ_pump_ = 585 nm, λ_probe_ = 406 nm, and θ = 55°) along with a fit to the curve (blue dashed), as well as the FT amplitude spectra (middle) and the short-time window Fourier transform (STFT) spectrograms (right) of the experimental oscillations obtained at different ion trap temperatures, (A) 270 K, (B) 200 K, (C) 150 K, (D) 100 K, (E) 50 K, and (F) 20 K. The fit parameters are given in Table S2. The STFT spectrograms are obtained by applying a 5.5 ps window time, providing a frequency resolution of 6 cm^*–*1^. The initial guess frequencies are shown as white dotted lines.

The effect of varying the ion-trap temperature can be better observed in the FT spectra given in the middle panel in Figure 5. Both FT spectra obtained at 20 and 50 K (Figure 5F,E) show well-resolved bands centered at 148, 108, 76, and 40 cm^–1^, as well as a weak high-frequency feature at 210 cm^–1^. In contrast, the FT spectra measured at 100 and 150 K (Figure 5D,C) exhibit only two dominant bands at 148 and 108 cm^–1^. The FT spectra measured at even higher temperatures (Figure 5B,A) do not resolve obvious frequency bands. We attribute this to the increasingly rapid dephasing of vibrational wave packets at higher ion internal temperatures, which can be more apparent in the STFT spectrograms given in the right panel of Figure 5. For example, the 148 cm^–1^ band persists for more than 5 ps in the STFT spectrogram at 20, 50, and 100 K (see Figure 5D–F), while it is limited to 4 ps at 150 and 200 K. At 270 K, all frequency bands decay within only 2 ps window time. At the highest temperature (270 K), bipyramidal Cu_5_^–^ can be thermally populated by only ≈0.02%. However, the vibrational-level population of neutral Cu_5_ is notably nonequilibrium and governed by the Franck–Condon principle. The neutral bipyramidal structure **2** can be populated at the highest temperature 270 K; see Figure S11. However, no new frequency bands characteristic of bipyramidal structure **2** were observed at any of the present ion-trap temperatures. This indicates that higher temperatures (or other suitable conditions) are required to overcome a kinetic energy barrier for structural isomerization or efficiently populate bipyramidal structure **2**.

The oscillation amplitude (*A*), frequency (ω_e_), lifetime (τ_1/2_), and initial phase (φ) are fitted using cosine functions, see Table S2 for detailed parameters. The fitted curves are plotted as blue dashed lines in Figure 5, which agree well with experimental oscillations. Two beating frequencies (ω_e_), 148 and 108 cm^–1^, only show minor shifts (<3 cm^–1^) at higher temperatures.

The oscillation lifetime τ_1/2_ values of 148 and 108 cm^–1^ bands are given in Figure 6, exhibiting an inverse correlation with ion-trap temperatures. Specifically, τ_1/2_ for the 148 cm^–1^ band decreases remarkably from 16.3 ps at 20 K, over 8.5 ps at 100 K, and down to 1.6 ps at 270 K. Similarly, τ_1/2_ for the 108 cm^–1^ band decreases from 8.3 ps at 20 K and down to 1.5 ps at 270 K. Hence, an increase in the ion internal temperature promotes dephasing and reduces vibrational coherence lifetimes.

**Figure 6:**
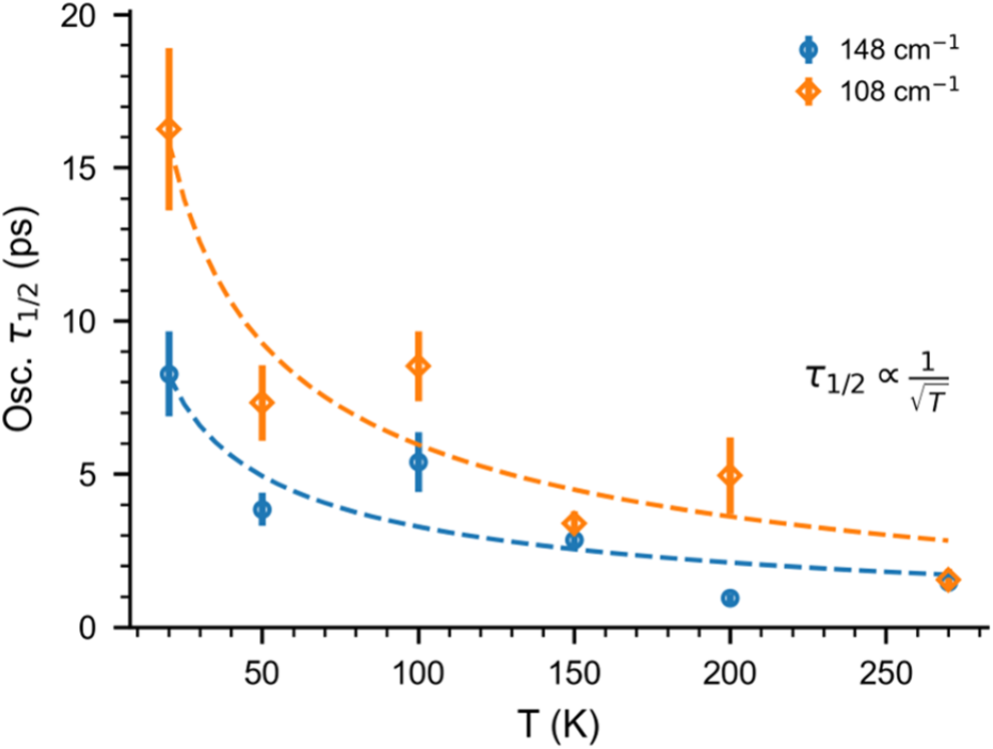
Temperature dependence of the oscillation lifetime (τ_1/2_) for two oscillation features at 148 and 108 cm^–1^, as well as the fitted curves (dashed lines) using the function $\tau_{1/2}=a\frac{1}{\sqrt{T}}+b$ . A comparison of the 1/ $\sqrt{T}$ and 1/*T* functions is given in Figure S12.

A more detailed interpretation of the ion-temperature influence on the wave-packet dynamics is provided below. We use two different functions, a 1/ $\sqrt{T}$ and a 1/*T* relation, to determine the oscillation lifetime. This is based on our simple assumption that the loss rate of the wave-packet oscillation (dWP/d*t*) is proportional to the number of the thermally populated high-lying vibrational levels (∑*N*_e_), given by

1 $$\frac{\mathrm{dWP}}{dt}\propto -\sum N_{e}$$

Accordingly, the oscillation lifetime, τ_1/2_, is inversely proportional to the total number of excited vibrational states, ∑*N*_e_.

Under the harmonic oscillator approximation, the vibrational energy of a single level is given by $E_{n}=\omega_{e}\left(n+\frac{1}{2}\right)$ . The total number of excited vibrational states can be approximated by the integral of the density of energy states (d*N*/d*E*) and the Boltzmann thermal population:

2 $$\int N_{e}\left(E\right)dE\;\propto \;\int g\left(E\right)e^{-E/k_{B}T}\;dE\;\propto \;T$$

where *g*(*e*) = 1/ω_e_ is the vibrational density of states, which is constant for a harmonic oscillator. This leads to a linear dependence of ∑*N*_e_ ∝ *T*, particularly for low-frequency modes and at high temperature, and consistent with the τ_1/2_ ∝ 1/*T* relation.

When anharmonicity is taken into account, the energy levels can be written as $E_{n}=\omega_{e}\left(n+\frac{1}{2}\right)-\;\chi_{e}\omega_{e}{\left(n+\frac{1}{2}\right)}^{2}$ . This introduces a nonuniform energy interval, causing the density of states to vary with energy, $g\left(E\right)=1/\sqrt{\omega_{e}-4\omega_{e}\chi_{e}E}$ , and leading to a limitation of vibrational energy asymptotic to the dissociation energy, *D*_e_ ≈ ω_e_/4χ_e_. One can find,

$$\int N_{e}\left(E\right)dE\;\propto \int \frac{1}{\omega_{e}\sqrt{1-E/D_{e}}}e^{-E/k_{B}T}\;dE\;\propto \;T^{1/2}$$ 3

This shows a slower growth of ∑*N*_e_ with temperature and suggests a weaker temperature dependence of vibrational damping, consistent with the τ_1/2_ ∝ 1/ $\sqrt{T}$ relation.

The comparison of the results obtained for two fit functions, representing the harmonic and anharmonic models, is given in Figure S12. The results for the 1/ $\sqrt{T}$ relation (Figure S12A) agree better than for the 1/*T* function with respect to the temperature dependence of τ_1/2_, particularly for the results at higher temperatures (>200 K). This indicates that inhomogeneous mechanisms such as anharmonicity play a significant role in the temperature dependence of wave packet dephasing.

The vibrational-level population of Cu_5_ is nonequilibrium, since it is prepared by photodetachment which is governed by the Franck–Condon factors and the excitation mechanisms. At the lowest temperature of the study (20 K), low-energy vibrational levels can be populated up to 1200 cm^–1^, yielding a set of (near-)­isoenergetic states (e.g., 2ν_4_ ≈ ν_2_, ν_4_ν_5_ ≈ ν_3_), see the computed populations of neutral Cu_5_ in Figures S9 and S11. The number of populated vibrational levels is still small at 20 K, but it becomes higher at 100 K. This rationalizes the two factors that our model is based on: (a) a high density of populated neutral vibrational levels arising from the low-frequency vibrational modes, which justifies the above integrals, and (b) the presence of a nonthermal population of these levels prepared by photodetachment, which leads to the population of high-quanta vibrational levels, except at low temperatures (<100 K) where the vibrational states are sparse.

Population of high-quanta vibrational levels might potentially contribute to inhomogeneous dephasing resulting from intramode anharmonicity. The Cu_5_ is isolated in the gas phase and within a cryogenic environment, which does not contain any surrounding thermal “bath” states. Thus, the additional decoherence channels in the system may involve other weakly coupling yet densely spaced vibrational states that are not probed. The current method does not allow to distinguish between these two mechanisms. In contrast, recent progress in two-dimensional infrared spectroscopy of trapped ions overcomes the limitation by exploiting diagonal and off-diagonal signatures.^65–68^

## 4 Conclusions

The vibrational wave-packet dynamics and frequency analysis of the fs NeNePo transients of Cu_5_ obtained at 20 K revealed frequencies at 222, 216, 148, 108, 76, and 40 cm^*–*1^ contributing to the observed quantum beats. Based on a comparison to DFT calculations, the beating frequencies originate from the electronic ground state (^2^A_1_) of planar Cu_5_. Unlike our previous study of Ag_5_, Cu_3_^+^ shows more pronounced oscillatory signals compared to that of Cu_5_^+^.

Temperature-dependent experiments up to 270 K showed no evidence of the presence of bipyramidal Cu_5_. Moreover, the oscillatory lifetimes decrease significantly with increasing temperature. This is likely due to two factors, anharmonicity of the populated highly lying vibrational states as well as the intramolecular vibrational energy redistribution (IVR) involving weakly coupled modes. This highlights the importance of low temperature for observing vibrational coherence.

The present results establish femtosecond NeNePo spectroscopy as a powerful tool for probing the vibrational wave-packet dynamics of neutral polyatomic metal clusters at variable ion-trap temperatures, in particular under conditions approaching ambient temperatures. This will be particularly useful for probing the dynamic properties of fluxional metal clusters under reactive environments, where higher temperatures are usually required.

## Supplementary Material



## Notes

Quadrupole mass spectra, laser power dependence measurements, fs NeNePo spectra, calculated vibrations, as well as detailed analysis of temperature effects (PDF)

## †

Department of Chemistry, State Key Laboratory of Porous Materials for Separation and Conversion, Shanghai Key Laboratory of Molecular Catalysis and Innovative Materials, Fudan University, Songhu Road 2005, 200438 Shanghai, China
