# Supplementary material for: Temperature Dependence of the Vibrational Wave-Packet Dynamics of Cu5
Source: J Phys Chem A. 2025 Nov 18;129(47):10873–82. doi: 10.1021/acs.jpca.5c06058 (PMC12670499; doi:10.1021/acs.jpca.5c06058)
Supplement: Supplementary file 1 [file jp5c06058_si_001.pdf]

Supporting information for 'Temperature Dependence of the Vibrational Wave-Packet Dynamics of Cu_5'

Jia Han,[†] Björn Bastian,[†] Marcel Jorewitz,[†] Knut R. Asmis,[*,†] and Jiaye Jin[*,†,‡]

†*Wilhelm-Ostwald-Institut für Physikalische und Theoretische Chemie, Universität Leipzig, Linnéstr. 2, 04103 Leipzig, Germany.*

‡*Present address: Department of Chemistry, State Key Laboratory of Porous Materials for Separation and Conversion, Shanghai Key Laboratory of Molecular Catalysis and Innovative Materials, Fudan University, Songhu Rd. 2005, 200438 Shanghai, China.*

E-mail: knut.asmis@uni-leipzig.de; jyjin@fudan.edu.cn

Contents

1 Fragments **S2**

2 Laser power **S3**

3 Fs NeNePo Spectra and frequency analysis **S5**

4 Calculated vibrational frequencies **S7**

5 Ion-Trap Temperature **S11**

1 Fragments

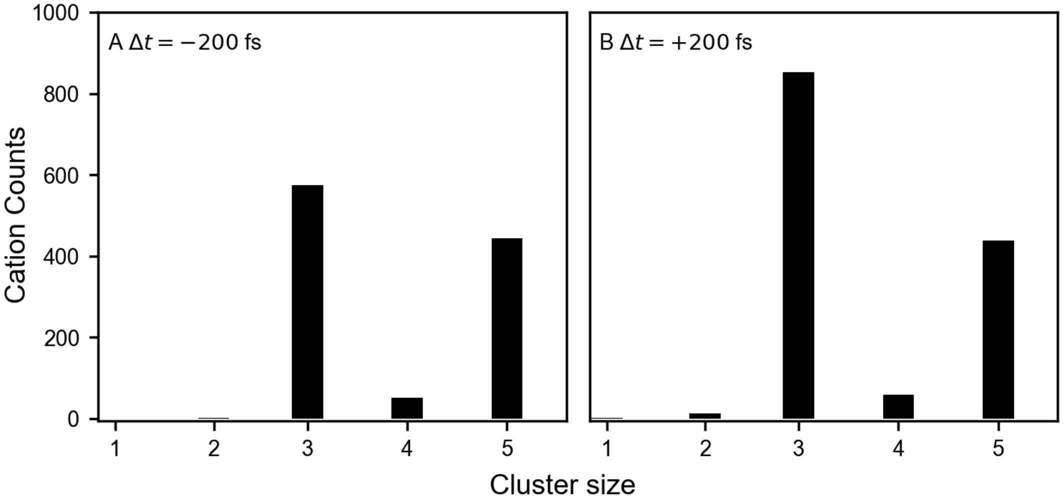

Figure S1: Integrated counts of Cu_n^+ ($n = 1 - 5$) from quadrupole mass spectra (1000 ms accumulation time) obtained at $\lambda_{\mathrm{pump}} = 585$ nm and $\lambda_{\mathrm{probe}} = 406$ nm: (**A**) delay time = -200 fs; (**B**) delay time = $+200$ fs.

2 Laser power

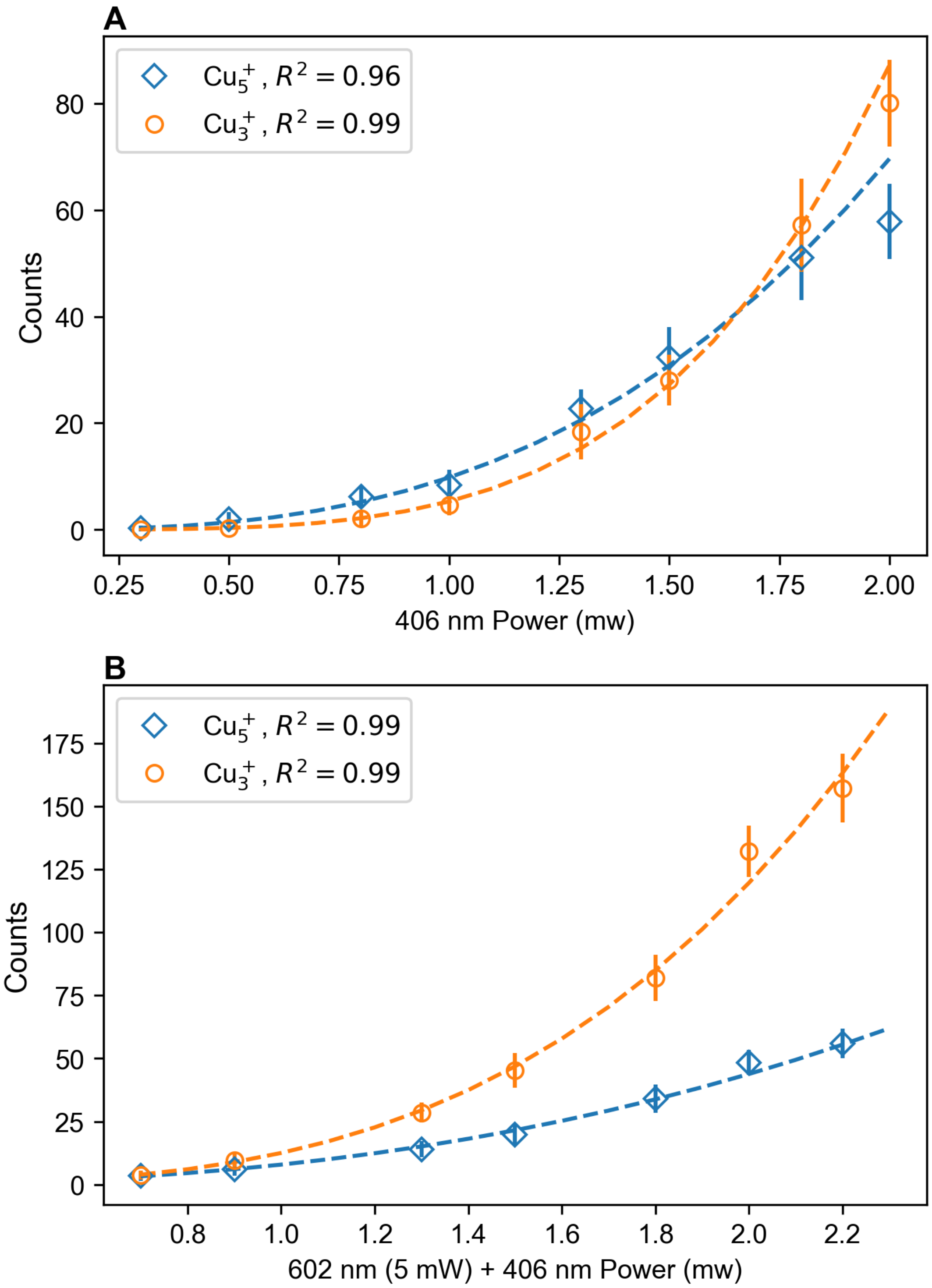

Figure S2: $Cu_5{}^+$ and $Cu_3{}^+$ counts as a function of laser power with fitted curves (dashed lines). (**A**): Single probe pulse (406 nm). (**B**): Probe pulse centered (406 nm) at a delay of 960 fs relative to the pump pulse (602 nm, 5 mW). See Figure S3 and S4 for fitting details.

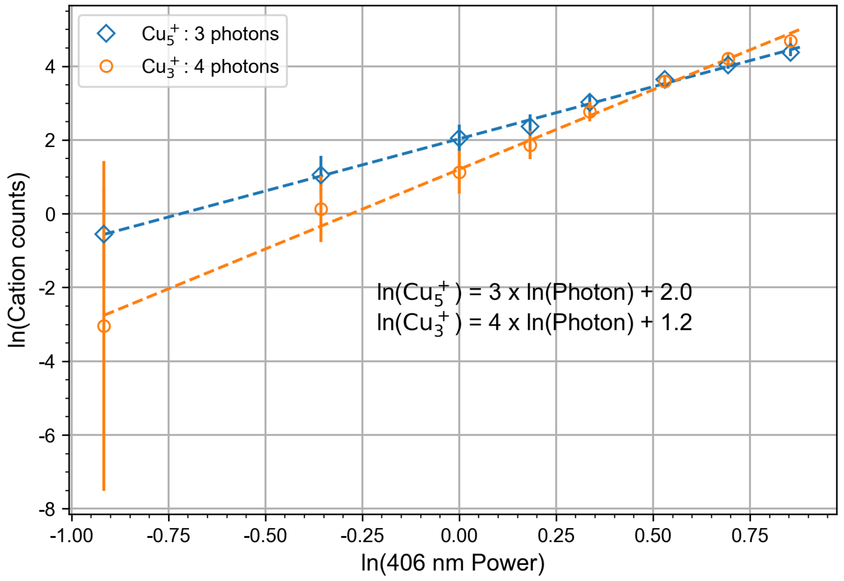

Figure S3: ${Cu_5}^+$ and ${Cu_3}^+$ counts as a function of probe power for a single probe pulse centered at 406 nm. The required photon order (n_{photon}) for NeNePo excitation is obtained from power-law fits, $ln(n_{cation}) = n_{photon} * ln(P_{laser}) + c$. Here, n_{cation} and P_{laser} are cation counts and the probe power (mW) measured behind the instrument, respectively. c is a fitting constant.

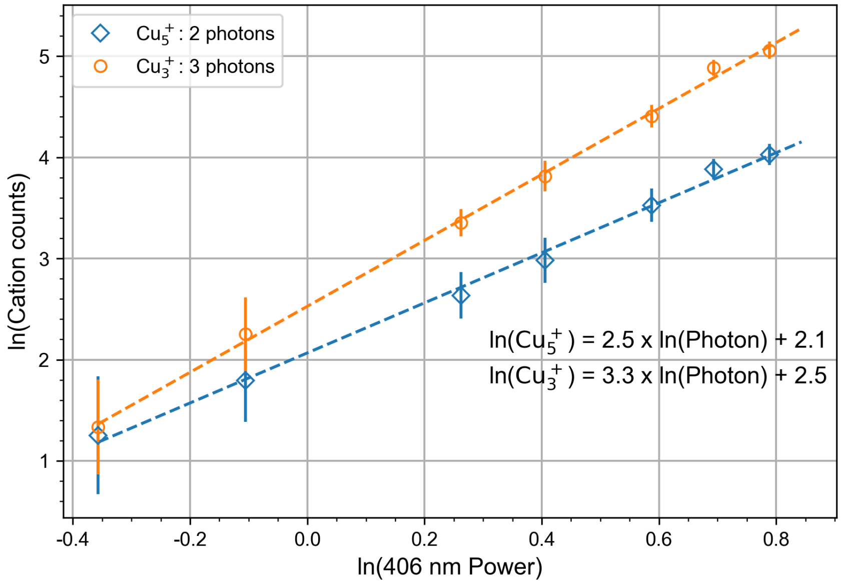

Figure S4: Two-color ${Cu_5}^+$ and ${Cu_3}^+$ counts as a function of probe power for the probe pulse centered at 406 nm, which is set at a delay of 960 fs relative to the pump pulse (602 nm, 5 mW). The required photon order (n_{photon}) for NeNePo excitation is obtained from power-law fits, $ln(n_{cation}) = n_{photon} * ln(P_{laser}) + c$. Here, n_{cation} and P_{laser} are cation counts and the probe power (mW) measured behind the instrument, respectively. c is a fitting constant.

3 Fs NeNePo Spectra and frequency analysis

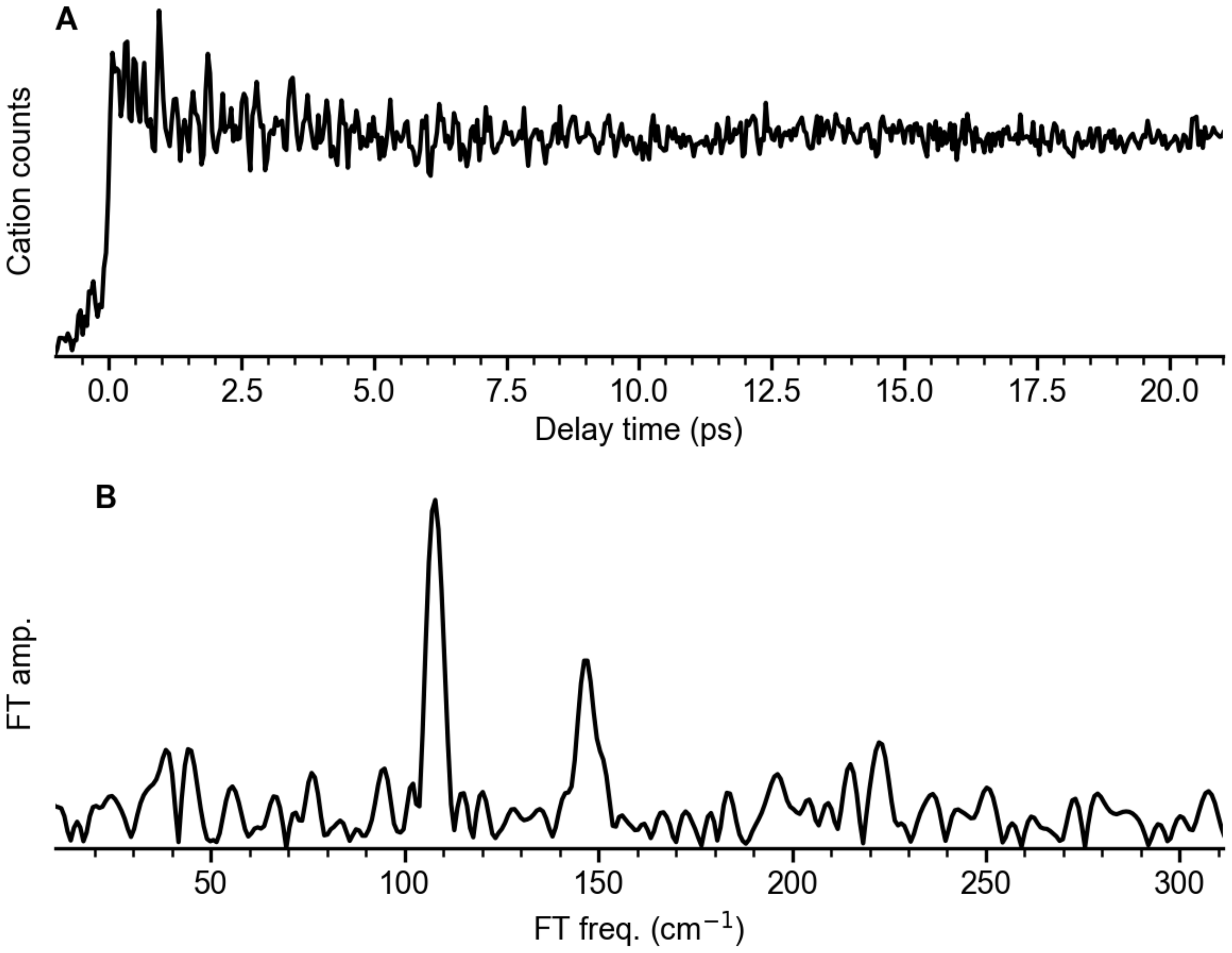

Figure S5: (**A**) fs NeNePo transients over a delay time range from −1 to 21 ps (λ_{probe} = 406 nm, λ_{pump} = 600 nm, polarization = 90°) and (**B**) Fourier transform (FT) amplitude spectrum for the oscillatory signal.

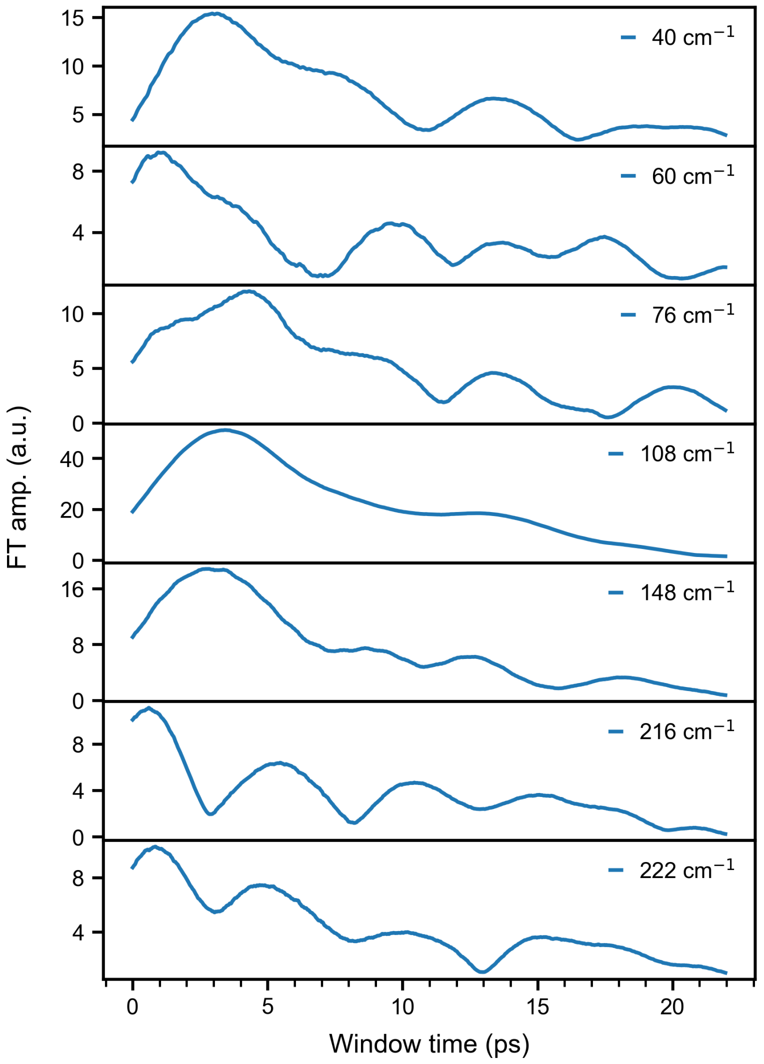

Figure S6: Short-time Fourier transform (STFT) amplitude vs window time for each principal bands centered at 40, 60, 76, 108, 148, 216, and 222 cm^{-1}. Traces correspond to the cuts along the dashed lines shown in Figure 3.

4 Calculated vibrational frequencies

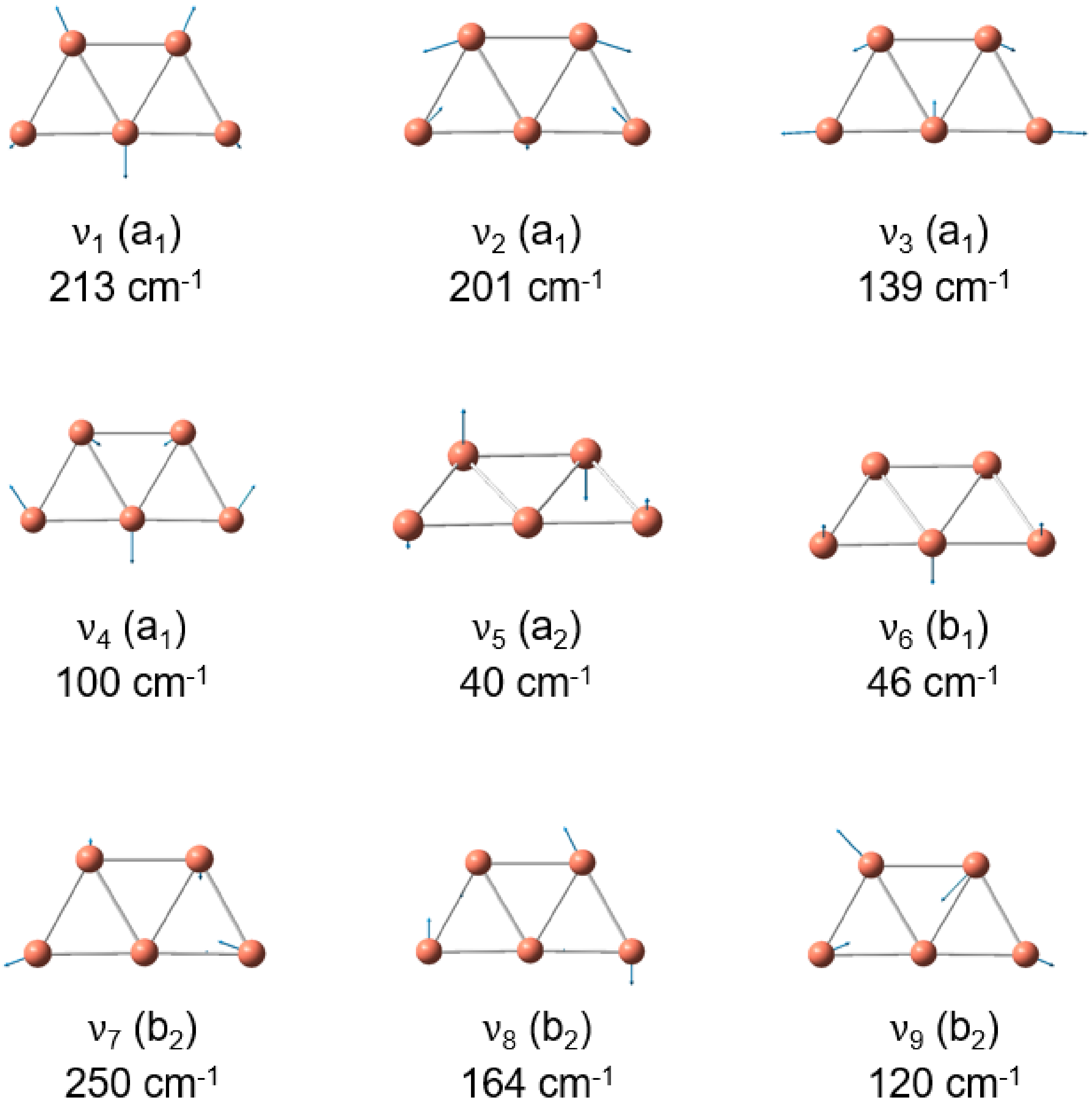

Figure S7: Normal modes of planar structure **1** and harmonic vibrational frequencies.

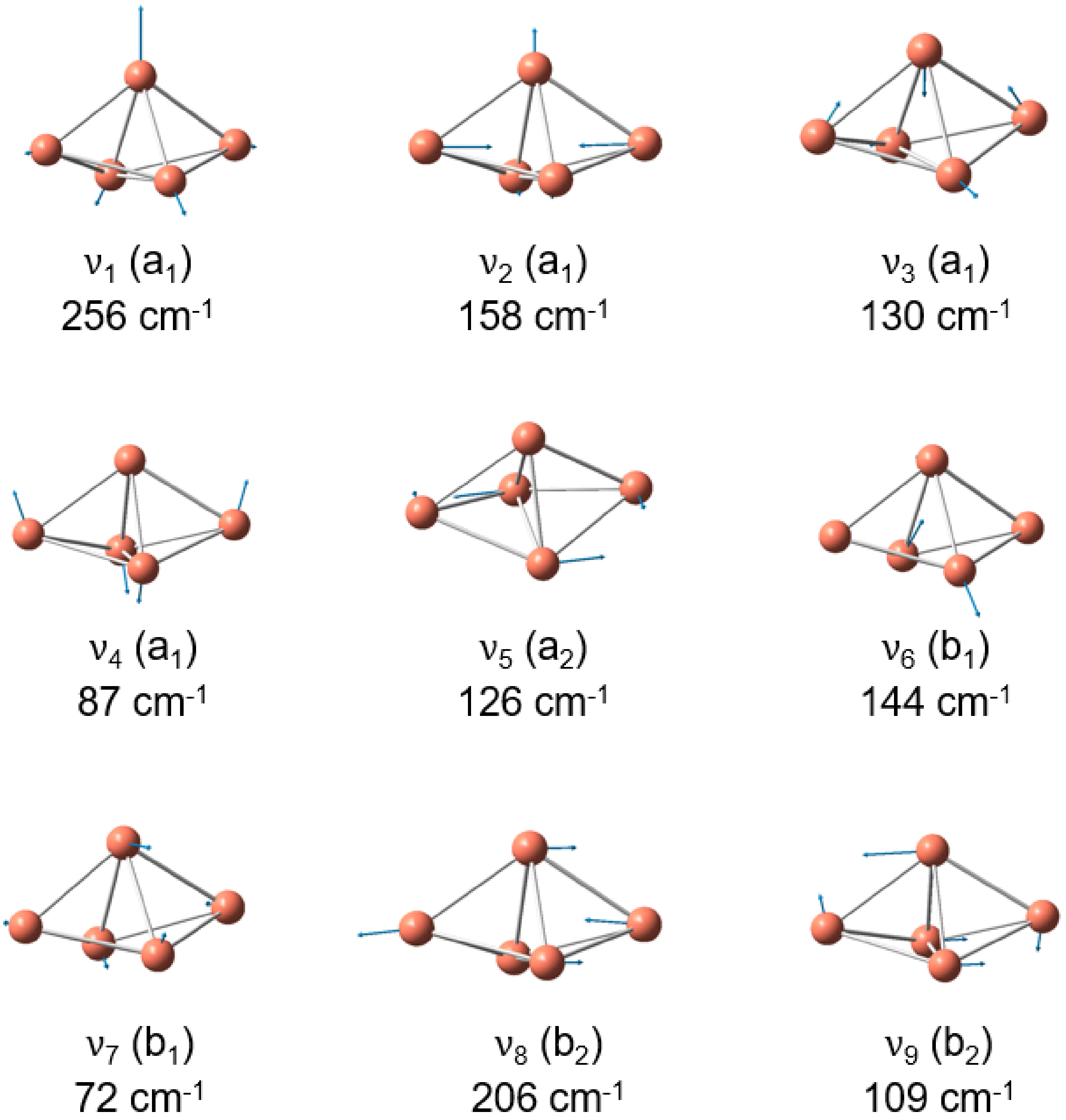

Figure S8: Normal modes of bipyramidal structure **2** and harmonic vibrational frequencies.

Table S1: Comparison of experimental FT frequencies and PBE0/def2-TZVPP harmonic frequencies (in cm^{-1}) of totally symmetric vibrational modes of Cu_5 and Cu_5^-. The normal modes are shown in Figure 4, S7 and S8.

Exp.		**1**			**2**	
	Neutral	Anion	Ass.	Neutral	Anion	Ass.
				256	233	$\nu_1(a_1)$
222	213	213	$\nu_1(a_1)$			
216	201	179	$\nu_2(a_1)/2\nu_4$			
148	139	128	$\nu_3(a_1)$	158	161	$\nu_2(a_1)$
				130	143	$\nu_3(a_1$
108	100	91	$\nu_4(a_1)$			
76			ν_1-ν_3	87	87	$\nu_4(a_1)$
60[a]			ν_2-ν_3			
40			ν_3-ν_4			

[a] Observed in the STFT spectrogram in the window time range from 0.1 ps to 3 ps, shown in Figure 3.

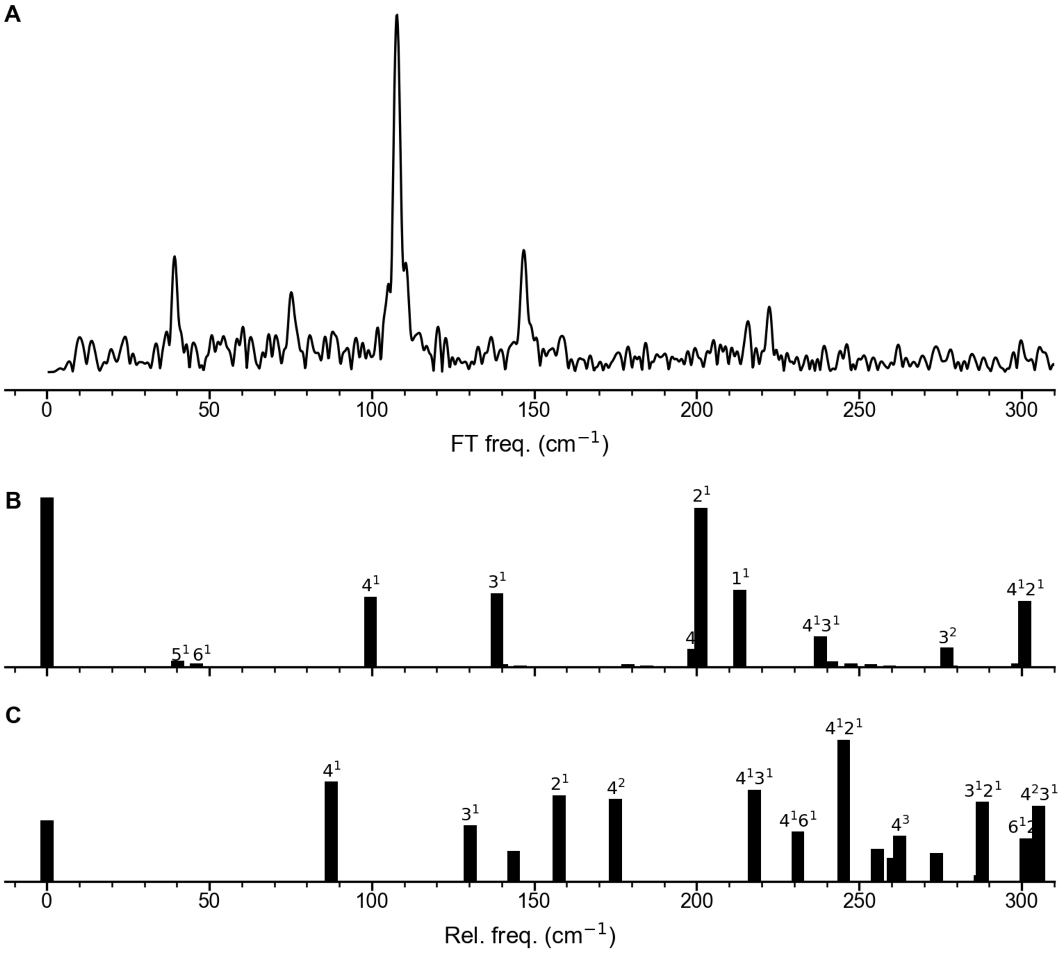

Figure S9: FT amplitude spectrum for $\lambda_{\mathrm{pump}} = 585$ nm (**A**) and computed population for the vibrational levels determined from Franck-Condon factors at 20 K for (**B**) the $\mathrm{Cu_5}\ (^2\mathrm{A_1}, \mathrm{C_{2v}}) \leftarrow \mathrm{Cu_5^-}\ (^1\mathrm{A_1}, \mathrm{C_{2v}})$ photodetachment transtion for planar structure **1** and (**C**) the $\mathrm{Cu_5}\ (^2\mathrm{B_1}, \mathrm{C_{2v}}) \leftarrow \mathrm{Cu_5^-}\ (^3\mathrm{A}_1', \mathrm{D_{3h}})$ photodetachment transtion for bipyramidal structure **2**. Normal modes are given in Figure S7 and S8.

5 Ion-Trap Temperature

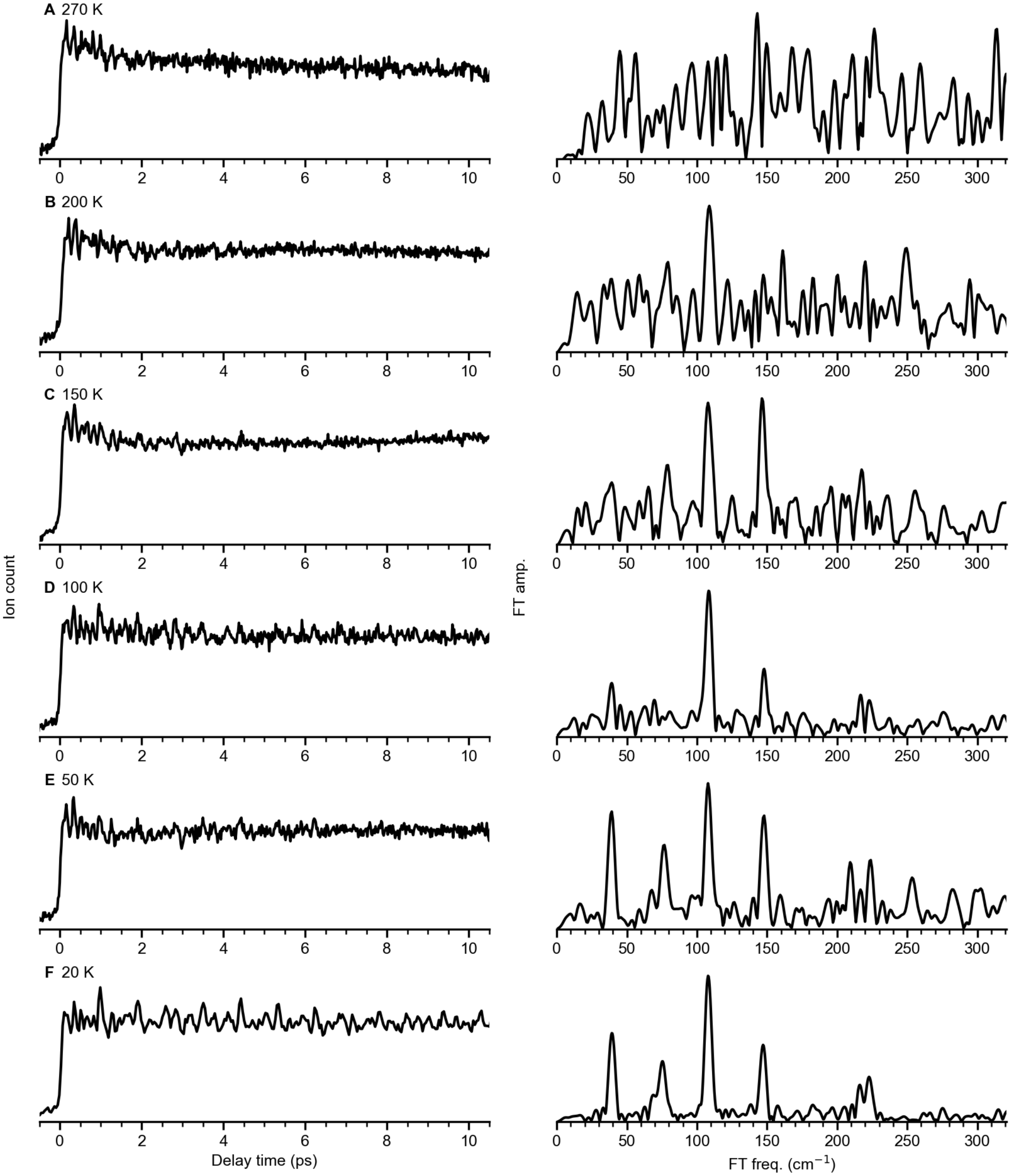

Figure S10: Fs NeNePo transients (left) as a function of pump-probe delay from −1 to 10 ps ($\lambda_{\text{probe}} = 405$ nm, $\lambda_{\text{pump}} = 584$ nm, $\theta = 55°$) and corresponding FT amplitude spectra (right) of the oscillatory signals obtained at different ion-trap temperatures, (**A**): 270 K, (**B**): 200 K, (**C**): 150 K, (**D**): 100 K, (**E**): 50 K, (**F**): 20 K.

Table S2: Fit parameters of the frequency contributions of the oscillatory signals shown in Figure 5 obtained from, $f(t) = \Sigma_i A_i cos(2\pi c\omega_{e,i}t + \varphi_i)e^{-t/\tau_i}$. A is the amplitude, φ the initial phase, τ the oscillation lifetime, ω_e the oscillation frequency, and c the speed of light.

T (K)	ω_e (cm^{-1})	φ (π)	A (a.u.)	τ (ps)
270	220	1.3	12	0.9
	207	0.1	6	06
	146	1.1	11	1.5
	111	5.6	9	1.6
	72	1.5	9	0.7
	53	1.4	7	1.8
200	215	1.1	21	1.6
	209	1.2	42	0.4
	145	0.8	37	1.0
	108	5.5	11	5.0
	76	0.9	12	2.9
	38	2.1	3	12.4
150	214	1.4	73	0.7
	237	0.1	22	0.4
	146	0.8	30	2.9
	108	5.6	27	3.3
	77	1.2	11	4.5
	39	1.6	10	4.7
100	216	1.5	36	1.5
	204	0.1	5	2.2
	147	1.2	20	5.4
	108	5.7	30	8.5
	69	1.9	16	3.7
	40	1.6	25	3.0
50	220	1.2	20	1.0
	198	0.2	24	0.5
	147	1.0	18	3.9
	108	5.7	15	7.3
	75	1.7	7	4.7
	39	1.7	9	10.9
20	220	1.1	64	1.2
	210	0.1	3	1.8
	147	1.1	30	8.3
	108	5.6	45	16.3
	76	1.6	30	6.7
	39	1.7	35	9.8

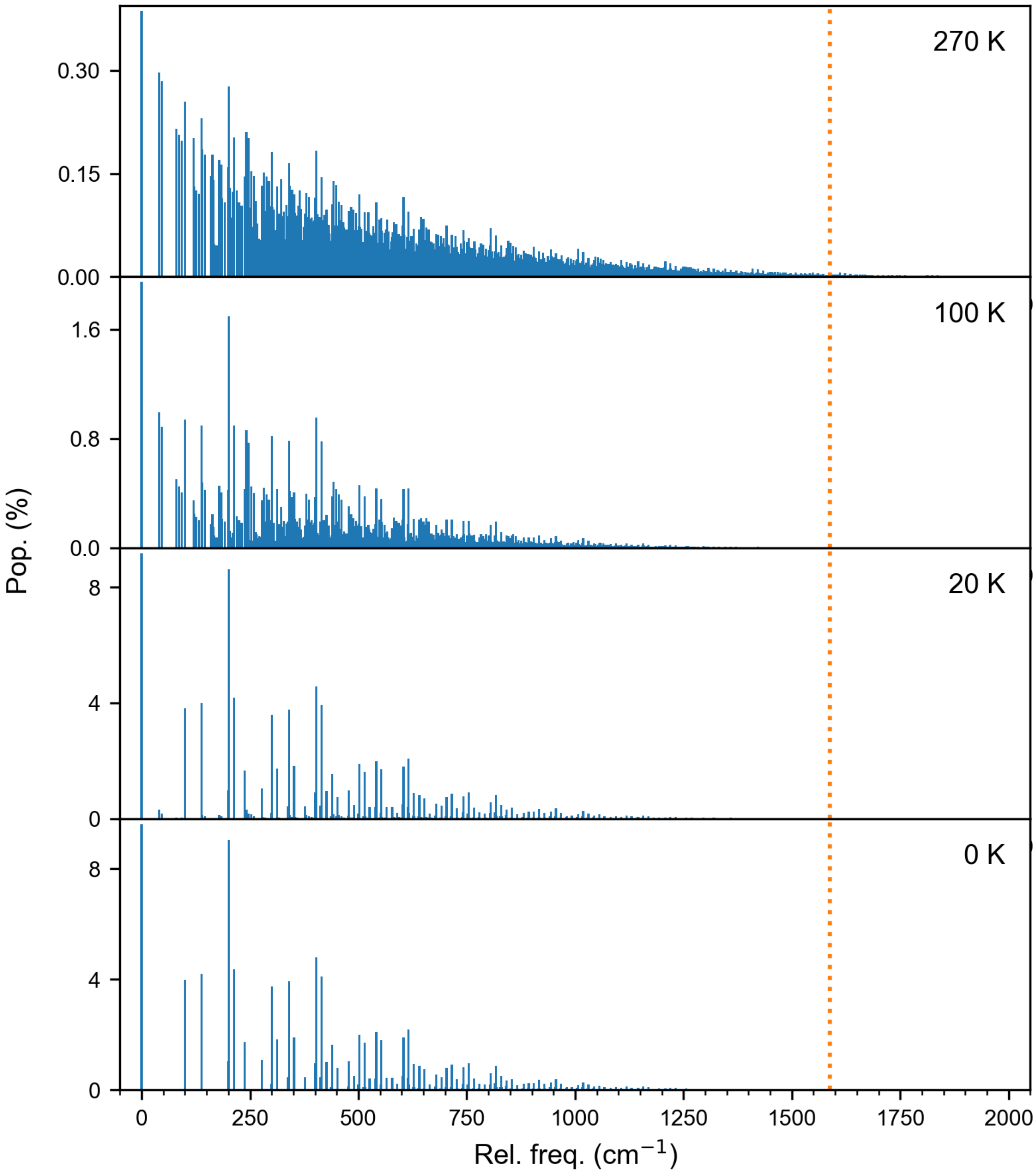

Figure S11: Computed population for the vibrational levels determined from Franck-Condon factors for the Cu_5 ($^2A_1, C_{2v}$) $\leftarrow$ Cu_5^- ($^1A_1, C_{2v}$) photodetachment transtion for planar structure **1** at different temperatures. The orange dashed lines mark the calculated energy of bipyramidal structure **2**, ≈ 1588 cm^{-1}.

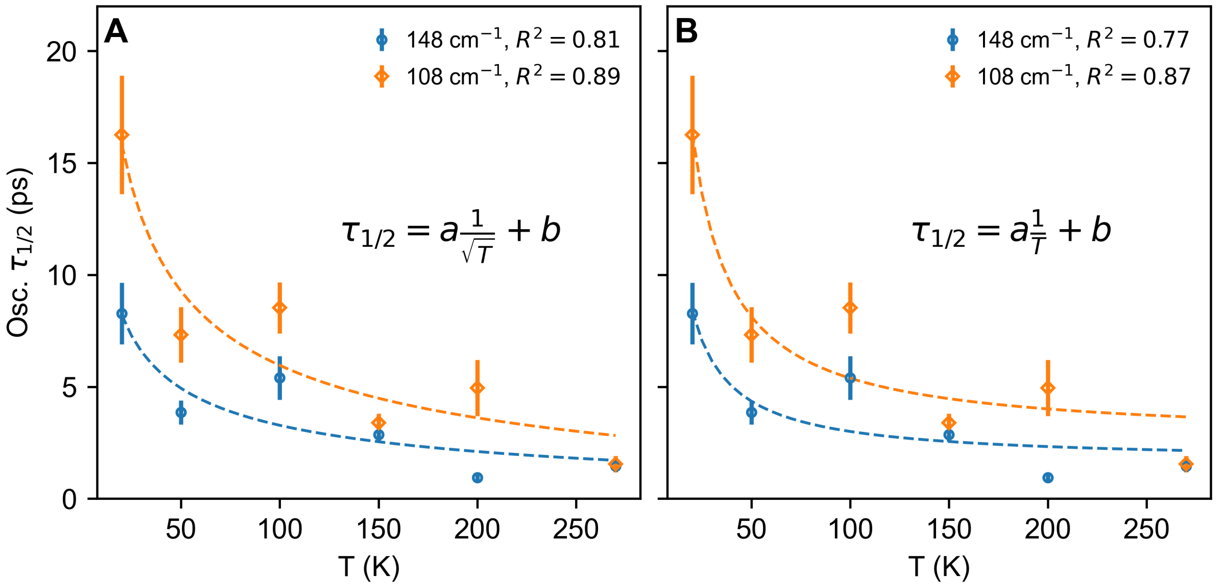

Figure S12: Comparison of fit functions applied to the ion-trap-temperature (T) dependence of the oscillation lifetime ($\tau_{1/2}$) for two features at 148 cm^{-1} and 108 cm^{-1}. Blue and orange dashed lines show the respective fit results, along with their R^2 values. (**A**): $\tau_{1/2} = a\frac{1}{\sqrt{T}} + b$; (**B**): $\tau_{1/2} = a\frac{1}{T} + b$.
